# An Eye-Tracking Study of Attention Biases in Children at High Familial Risk for Depression and Their Parents with Depression

**DOI:** 10.1007/s10578-020-01105-2

**Published:** 2021-01-04

**Authors:** B. Platt, A. Sfärlea, C. Buhl, J. Loechner, J. Neumüller, L. Asperud Thomsen, K. Starman-Wöhrle, E. Salemink, G. Schulte-Körne

**Affiliations:** 1 Department of Child and Adolescent Psychiatry, Psychosomatics and Psychotherapy, LMU University Hospital, Nußbaumstr. 5a, 80336 Munich, Germany; 2 Department of Clinical Psychology and Psychotherapy, LMU, Munich, Germany; 3 Department of Clinical Psychology, Utrecht University, Utrecht, The Netherlands

**Keywords:** Depression, Youth, Attention bias, Eye-tracking, Transgenerational, Parent

## Abstract

Attention biases (AB) are a core component of cognitive models of depression yet it is unclear what role they play in the transgenerational transmission of depression. 44 children (9–14 years) with a high familial risk of depression (HR) were compared on multiple measures of AB with 36 children with a low familial risk of depression (LR). Their parents: 44 adults with a history of depression (HD) and 36 adults with no history of psychiatric disorder (ND) were also compared. There was no evidence of group differences in AB; neither between the HR and LR children, nor between HD and ND parents. There was no evidence of a correlation between parent and child AB. The internal consistency of the tasks varied greatly. The Dot-Probe Task showed unacceptable reliability whereas the behavioral index of the Visual-Search Task and an eye-tracking index of the Passive-Viewing Task showed better reliability. There was little correlation between the AB tasks and the tasks showed minimal convergence with symptoms of depression or anxiety. The null-findings of the current study contradict our expectations and much of the previous literature. They may be due to the poor psychometric properties associated with some of the AB indices, the unreliability of AB in general, or the relatively modest sample size. The poor reliability of the tasks in our sample suggest caution should be taken when interpreting the positive findings of previous studies which have used similar methods and populations.

**Supplementary Information:**

The online version of this article (10.1007/s10578-020-01105-2) contains supplementary material, which is available to authorized users.

## Introduction

### Prevalence of Depression

Depression is one of the most common psychiatric disorders with a lifetime prevalence of around 20% [1]. It affects more than 300 million people worldwide [2] and is expected to be the leading cause of disability by 2030 [3]. Developing effective preventive interventions which target groups at risk of developing depression is of paramount importance if the burden of depression is to be reduced [4].

### Children of Depressed Parents: A High-Risk Group

One of the biggest risk factors for depression is having a parent who is suffering or has suffered from depression. Children of depressed parents are around three times as likely to develop depression as children of non-depressed parents [5], with up to 40% of children of depressed parents having their first episode by age 20 [6]. According to the most prominent transgenerational model of depression, biological (e.g., genetic), neuroregulatory (e.g., dysfunctional neuroregulation during pregnancy) and psychological (e.g., exposure to maladaptive parental coping strategies) factors render children of depressed parents more susceptible to environmental stress [7]. Understanding more about how these factors interact with each other may improve the development of preventive interventions.

### Attention Biases (AB) in Depression

One means by which biological, neuroregulatory and psychological vulnerability factors for depression may interact is via biases in attention processing. Negative attention bias (AB) refers to the tendency of depressed patients to preferentially attend to negative versus neutral or positive information [8]. For example, when confronted with a crowd of new faces at a party, a person with depression is more likely to dwell on the person frowning rather than the person smiling at them. Cognitive models of depression identify negative AB as a key characteristic of depression [8–10]. Since negative AB is influenced by genetic and neuroregulatory factors and is bi-directionally associated with psychological processes such as emotion regulation, they are proposed to play a key role in bridging the gap between biological, neuroregulatory and psychological vulnerability for depression [8].

### Measures of AB

Numerous behavioral and eye-tracking (ET) tasks have been developed to measure AB [11]. The most prominent behavioral measure of AB is the modified Dot-Probe Task (DPT) [12].^1^ In this task an emotional (e.g., negative) and a neutral stimulus (commonly faces) are displayed simultaneously for 500–1500 ms, followed by a brief probe that appears on the side of the negative (congruent) or neutral (incongruent) stimulus. Reaction times (RTs) to identify a characteristic of the probe (e.g., vertical versus horizontal presentation) are measured. Faster RTs on congruent trials indicate an AB towards negative information, whereas faster reaction times on incongruent trials indicates an AB away from negative information. The reliability (internal consistency) of the DPT has been called into question, with unacceptable reliability estimates^2^ reported in some adult [13–21] and youth [19, 22] samples. An additional behavioral measure of AB is the modified Visual-Search Task (VST) [23], which measures the speed at which participants are able to detect a target stimulus (e.g., happy face) in the presence of emotional distractors (e.g., sad faces). The VST has shown acceptable internal consistency in adults [18, 19, 24]. The apparent superior reliability of the VST over the DPT may be because stimuli are exposed for longer than in the DPT and the proportion of task:error variance may therefore be greater in the VST. It should however be noted, that since the majority of AB studies to date have not reported the psychometric properties of the tasks in their samples, it is too soon to say whether the VST is categorically superior over the DPT. The internal consistency of the VST in youth samples is unclear: internal consistency for children aged 7–9 years falls within the “unacceptable” range [19, 22] (*REL* < .5) but is nevertheless higher than the DPT. It is unknown how reliable the task is for children over 9 years.

RT-based measures of AB such as the DPT have a number of additional methodological limitations [25]. Firstly, as attention is inferred from a participant’s single response, RT-based measured cannot capture the dynamic nature of attention across time or distinguish between multiple sub-components of attentional processing such as orientation of attention, maintenance of attention, and attentional avoidance [26]. Note that modified DPT paradigms [27] and alternative analysis strategies, e.g., Attention Bias Variability (ABV) and Trial Level Bias Score (TL-BS), have been developed to address this issue. Nevertheless, these have also been met with criticism since they may not be able to differentiate between measurement error and bias variability [28]. Furthermore, behavioral measures such as the DPT are also affected by participants’ motor responses, which may be a particular problem for investigating depression which is accompanied by generally slowed motor responses [8].

Eye-tracking (ET) has the advantage of being able to distinguish between the various sub-components of AB such as speeded *orientation to* negative stimuli (i.e., location of first fixation) and increased *maintenance* of attention on negative stimuli (i.e., dwell time on negative stimuli). In ET studies, a participant’s attentional processing is inferred from their eye-movements and direction, most commonly using Passive-Viewing Tasks (PVT), in which participants are shown a variety of emotional stimuli at once without instruction. This task allows continuous assessment of the visual attention during relatively long free-viewing trials. The PVT is not affected by motor responses and has been associated with excellent internal consistency and acceptable one-week test–retest reliability [29].

### Evidence of AB in Adult and Youth Depression

Meta-analyses of behavioral AB studies in adult populations suggest between-group differences (depressed versus non-depressed) of a medium^3^ effect size (ES) [30, 31]. A meta-analysis of ET studies found medium to large ESs for between-group differences in dwell-time on negative and positive stimuli but no evidence of a negative AB in the initial orientation of attention [32]. Preliminary evidence from experimental [33–35] and longitudinal [36, 37] studies supports the causal role of negative AB in the development and maintenance of depression [9], perhaps by impairing emotion regulation [38, 39].

Models of the etiology of youth depression also posit AB to play a key role [40] although the empirical basis for this is less established than for adults [41]. Individual cross-sectional behavioral (largely DPT) studies have found AB towards negative (sad or threatening) stimuli to characterize depressed but not non-depressed youth [42–47]. Note that some earlier studies failed to observe such group differences, but this may be due to their relatively modest sample sizes [48, 49]. A single ET study of youth depression found *reduced* dwelling on negative stimuli, but these findings are yet to be replicated [50]. The extent to which AB plays a *causal* role in youth depression remains unclear [23, 51].

### AB in the Children of Depressed Parents

Negative AB might be passed from parent to child via polygenetic risk factors associated with the hypothalamic–pituitary–adrenal (HPA) axis [52]. Alternatively, or additionally, children of depressed parents might acquire a negative AB by modelling their parents’ negative reactions to ambiguous stimuli (“Look at all of those people ignoring us!” versus “Look at all of those people smiling at us!”) [53]. Nevertheless, only a small number of studies have investigated the nature of AB in the children of depressed parents. The majority of studies of children of depressed parents have used the DPT and found a negative AB *towards* sad [54, 55] and angry [56, 57] faces in the (healthy) children of depressed versus non-depressed mothers and fathers [58]. Two further DPT studies found evidence of an AB *away* from sad faces in the children of depressed versus non-depressed parents [59, 60]. Comparing the findings from these few studies is limited by numerous methodological differences between them. For example, children in the latter two studies showed elevated symptoms of depression and the parents in one of them had elevated self-reported symptoms of depression but did not meet the diagnostic criteria for a diagnosis of depression [59]. Some studies used a mood-induction procedure [54, 55] while others did not [56–60]. Mood induction procedures are particularly important in studies of non-clinical samples since diathesis-stress models of depression posit cognitive biases to only emerge under conditions of stress or low mood [61, 62]. The stimuli themselves also varied between studies, with some studies finding a negative AB is specific to sad (but not angry or happy) faces [60] and others finding AB with angry (but not happy) stimuli [56, 57]. The extent to which the heterogeneous findings are influenced by the psychometrics of the tasks employed is unclear since none of the studies report the internal consistency of the DPT in their sample. Just one has used ET, finding children of depressed (versus non-depressed) parents to spend significantly more time dwelling on sad stimuli with a medium to large ES [52].

### Studies of the Association between Parent and Child AB

A necessary next step in understanding the role of AB in the transgenerational transmission of depression risk is to test the extent to which parent and child AB are associated with each other. To date, just one study has examined AB in HR children and their mothers with depression or anxiety disorders [56]. This behavioral DPT study found that mothers with an emotional disorder who showed avoidance of positive stimuli were more likely to have children who showed an AB towards negative stimuli. However, these findings are limited to a sample of mothers with a lifetime history (not specific to the child’s lifetime) of any mood disorder (including anxiety).

### The Current Study

The present study tested three hypotheses: (i) that non-affected youth at high familial risk (HR) show more negative AB than non-affected youth at low familial risk (LR), (ii) that parents with a history of depression (HD) show more negative AB than those with no psychiatric history (ND), and (iii) that across the sample, youth AB are positively associated with parent AB.

To enable comparison with previous studies, we used the DPT. An additional behavioral measure which shows superior reliability was also used: the modified VST [23]. We ran the VST during ET due to predictions about the superior reliability of ET measures. In addition, a PVT was administered during ET to distinguish between attention orientation and maintenance. A negative mood induction was applied before administering the experimental tasks. AB was measured in relation to both angry and sad stimuli and was tentatively expected to be stronger for depression specific stimuli, i.e. sad than angry faces.

An additional aim of the study was to describe and evaluate the psychometric properties of the various measures of AB in our sample. Indeed, whereas it is common practice to report the psychometric properties of questionnaire-based measures, numerous researchers have highlighted the lack of reporting of reliability in behavioral and ET studies and the need for this to become routine if progress is to be made in the field [9, 63–65]. As Gibb et al. emphasize: “Because reliability places an upper limit on validity, establishing the reliability of one’s measures is essential to move the field forward.” [60, p. 11]. We expected the DPT to show the poorest reliability (internal consistency) and superior reliability in the VST and PVT measures. Previous studies suggest relatively poor convergent reliability between measures of AB [18] so this was assessed as well. Correlations of all measures of AB with depressive symptoms and anxiety scores were also explored to assess construct validity and the extent to which AB are specifically related to depressive symptoms.

## Methods

The present data on AB were collected within a broader project on cognitive biases in the offspring of parents with depression [66]. Data from AB tasks^4^ are presented here while data from tasks assessing interpretation biases (IB) are presented elsewhere [67].

### Participants

A total of 80 parent–child dyads were included in the data analysis.^5^ Of the parents, *n* = 44 had a history of depression (HD group) so their *n* = 44 participating children were considered to have a high risk for depression (HR group). The remaining *n* = 36 parents had no history of depression or any other mental disorder (ND group) so the corresponding *n* = 36 children were considered to have a low risk for depression (LR group). Children were 9–14 years old; children younger than 9 years were not included due to concerns about their ability to understand and perform the tasks. Adolescents older than 14 years were not included since the incidence of depression increases substantially after that age [68]. Examining older children of depressed parents that had not yet suffered from an episode of depression might result in examining a particularly resilient and therefore non-representative HR sample.

The sample size was based on an a priori power analysis (α error probability = .05; power = .8; two-tailed). Based on effect sizes from previous studies of AB in the children of depressed versus non-depressed parents that included mood induction procedures [54, 55], we expected an effect size of at least *d* = 0.7 for our main aim (comparing HR and LR children). This resulted in a required sample size of *n* = 68. Some of the HD/HR families were recruited through a study evaluating an intervention to prevent the development of depression in children of parents with a history of depression [69],^6^ while others as well as the ND/LR families were recruited via local advertisements, previous studies, and mailings to randomly-selected families with children in the corresponding age range provided by the local registry office.^7^

All participants underwent extensive diagnostic assessment before inclusion in the study. Standardized, semi-structured psychiatric interviews were administered to assess psychiatric diagnoses in parents (DIPS) [70] and children (K-DIPS; conducted with both child and parent) [71]. The DIPS and the K-DIPS are well-established German diagnostic interviews that allow diagnosis of a wide range of psychiatric axis I disorders according to DSM-IV [72] with good interrater-reliabilities (accordance rate of at least 87% was found for all diagnoses [73, 74]). The interviews were conducted and evaluated by trained interviewers and interrater-reliability in our study was determined for 20% of the sample by an independent researcher re-rating audio recordings of the diagnostic interviews. The accordance rate for lifetime diagnosis of depression (pre-defined criterion) was 94% for the DIPS and 100% for the K-DIPS.

Parents were included in the HD group if they met criteria for major depression (*n* = 42) or dysthymia (*n* = 2)^8^ during the child’s lifetime. Exclusion criteria were a history of bipolar disorder, psychosis, or substance abuse. Of the parents who met criteria for major depression, 34 had suffered recurrent episodes of major depression, and seven parents were currently depressed. More than half of the HD parents (*n* = 24) were taking psychotropic medication (mostly selective serotonin reuptake inhibitors) and almost all of them were either currently undergoing psychotherapy (*n* = 15) or had previously received at least some form of psychotherapy (*n* = 26). Regarding comorbidities, 12 parents currently met criteria for at least one other psychiatric disorder. Parents were included in the ND group if they did not meet criteria for any past or current axis I disorder. To ensure that neither of the parents in the ND/LR families had ever met criteria for a psychiatric disorder, psychiatric diagnoses and depression scores were also obtained from the second parent wherever possible.^9^ Children who did not meet criteria for any current or past axis I disorder^10^ and had an IQ ≥ 85 (assessed using the CFT 20-R [75]) were included in the study. Demographic and clinical characteristics of the child and parent samples are presented in Table 1. The parent groups were comparable in terms of age and gender ratio but differed significantly, as expected, regarding depression and anxiety symptoms.^11^ Groups of children did not differ significantly in terms of age, IQ, gender ratio or symptoms of depression or anxiety. All included participants had normal or corrected to normal vision.

**Table 1:** Demographic and clinical characteristics of the sample

	Children				Parents			
	HR	LR	Test statistics		HD	ND	Test statistics	
	*n* = *44*	*n* = *36*			*n* = *44*	*n* = *36*		
Gender m/f	18/26	13/23	*χ*^*2*^ < 1	n.s.	12/32	5/31	*χ*^*2*^ = 2.1	n.s.
Age; *M (SD)*	11.5 (1.5)	11.8 (1.7)	*t* < *1*	n.s.	46.3 (6.1)	45.0 (4.5)	*t*_77.3_ = 1.1	n.s.
IQ; *M (SD)*	108.6 (11.4)	112.5 (10.7)	*t*_78_ = *1.6*	n.s.	n.a.	n.a.		
Depression; *M (SD)*	7.4 (5.4)	5.6 (4.8)	*t*_77_ = *1.5*	n.s.	10.5 (8.3)	1.9 (3.6)	*t*_62.0_ = 6.1	*p* < *.001*
Anxiety; *M (SD)*	29.8 (6.4)	28.1 (6.3)	*t*_78_ = *1.2*	n.s.	44.6 (10.2)	31.0 (7.9)	*t*_76_ = *6.5*	*p* < *.001*

In children, depressive symptoms were assessed with the German version of the Children’s Depression Inventory (DIKJ; raw values presented) and anxiety was assessed with the trait scale of the State Trait Anxiety Inventory for Children (STAIC-T). In parents, depressive symptoms were assessed with the Beck Depression Inventory-II (BDI-II) and anxiety was assessed with the trait scale of the State Trait Anxiety Inventory (STAI-T); *HR* high-risk, *LR* low-risk, *HD* history of depression, *ND* never-depressed, *n.a.* not applicable, *n.s.* not significant

The study was approved by the institutional ethics committee. Written informed consent was obtained from all participants after a comprehensive explanation of the experimental procedures. Families received a reimbursement of €50 for participation.

### Experimental Tasks

#### Stimuli

In all three tasks, stimuli were colored photographs of faces displaying different emotional expressions that were presented on black background. Stimuli were age-matched, i.e., children viewed pictures of child models from the NIMH Child Emotional Faces Picture Set (NIMH-ChEFS) [76], while parents viewed pictures of adult models taken from the NimStim dataset [77]. Half of the models were male and half of the models were female in each task. For the DPT, 10 models per age-group showing angry, sad, and neutral faces were used. For the VST, photographs of 16 models per age-group displaying angry, sad, and happy emotional expressions were selected. For the PVT, the stimulus set comprised photographs of 24 models per age group displaying angry, sad, happy, and neutral emotional expressions.

#### Dot-Probe Task (DPT)

**Fig. 1:**
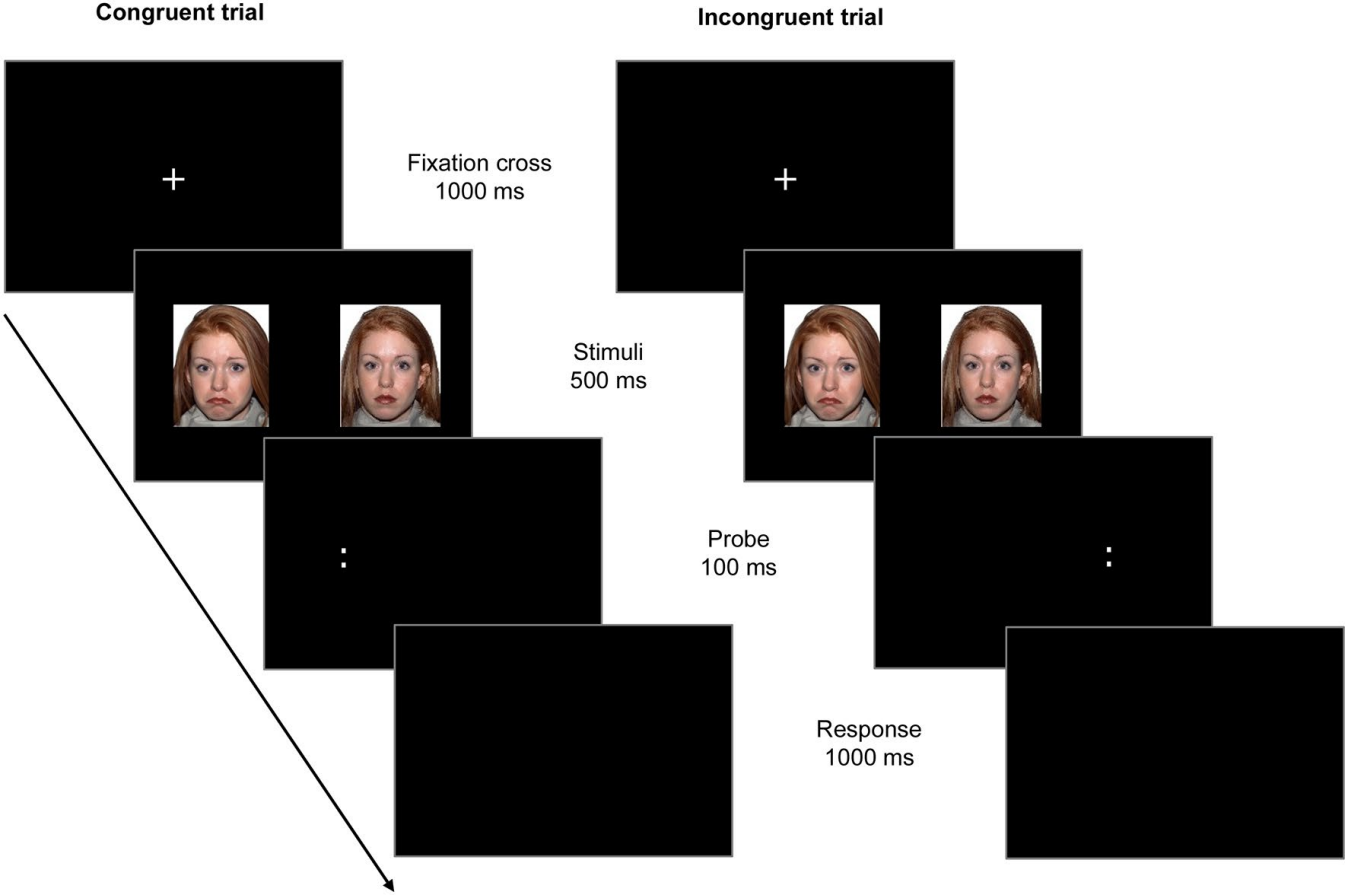
The adult version of the modified Dot-Probe Task (DPT) [12, 78]. Stimuli taken from the NimStim dataset [77]. Procedure corresponding to [51]

A modified version of the DPT [12, 51, 78] was used to assess AB for sad as well as angry faces via RTs (Fig. 1). Participants were seated in front of a 24-in. computer screen (1920 × 1080 pixel resolution) at a viewing distance of approximately 65 cm. The experiment was presented using E-Prime 2.0 [79]. Each trial started with a fixation cross that was presented for 1000 ms at the center of the screen. Then the face stimuli were presented for 500 ms. Two pictures of the same actor were presented side-by-side: either an emotional (sad or angry) expression paired with a neutral expression (emotional trials) or two neutral expressions (these neutral filler trials were not analyzed). Pictures had a size of approximately 13.5 × 10.5 cm and were presented with a distance of approximately 18 cm between them. The faces were followed by the probe which appeared in the location of one of the faces for 100 ms. The probe was two dots presented either vertically (“:”; in 50% of trials) or horizontally (“..”; 50% of trials) and participants were required to react as quickly and accurately as possible to the probe orientation by pressing one key for vertical and another key for horizontal orientation. The probe was followed by a blank screen presented for 1000 ms during which responses were recorded (i.e., within 1100 ms after probe onset).

The task comprised four blocks in random order: two measuring AB for sad faces and two measuring AB for angry faces. Each block consisted of 20 congruent trials (i.e., emotional trials in which the probe appeared in the location of the emotional face), 20 incongruent trials (i.e., emotional trials in which the probe appeared in the location of the neutral face), and 10 neutral trials, summing up to a total of 40 congruent and 40 incongruent trials per emotion across the whole task. Within each block, trials were presented in random order with emotional faces as well as the probe presented equally often on each side. Before the first block, participants completed sixteen practice trials in which they received feedback, in order to familiarize themselves with the task.

#### Visual-Search Task (VST)

**Fig. 2:**
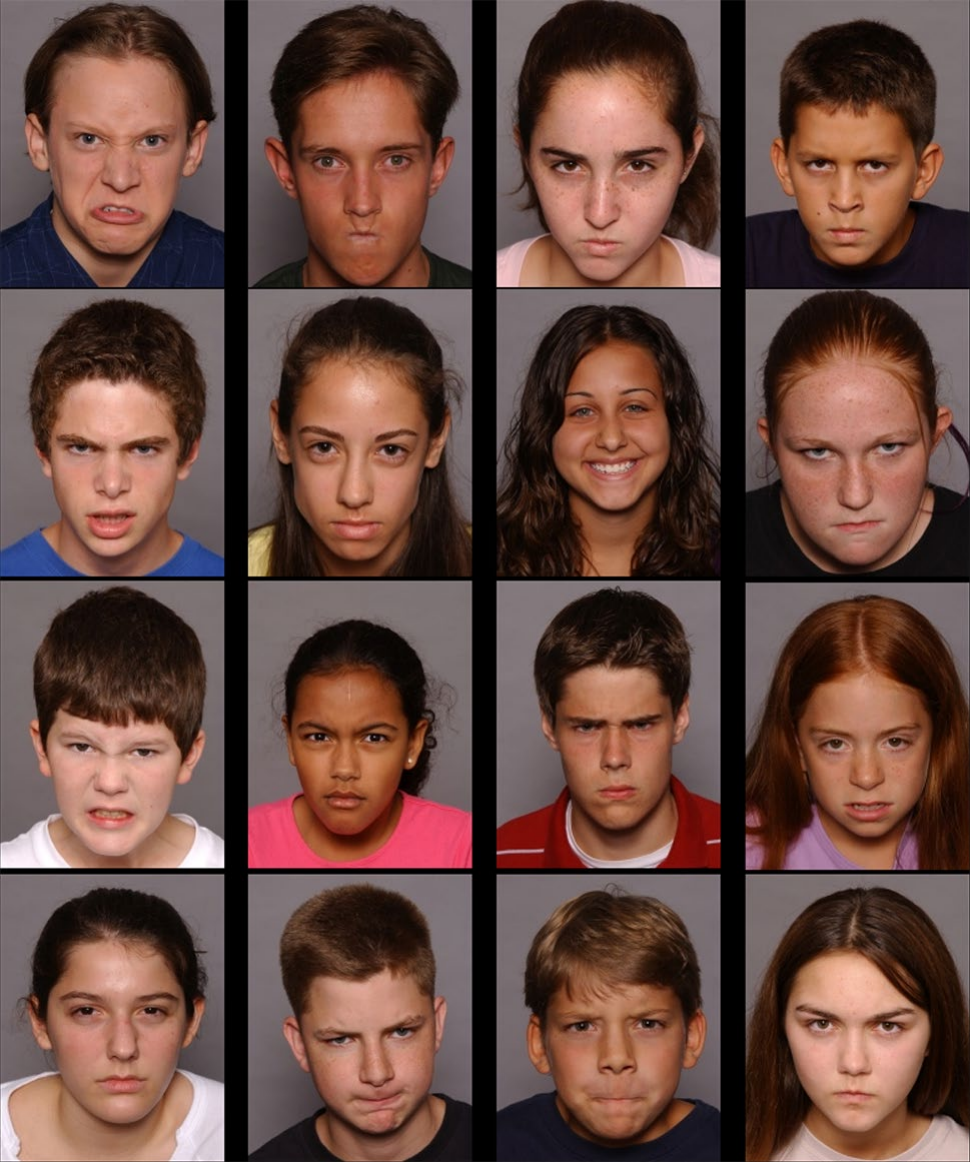
The children’s version of the Visual Search Task (VST) [23]. Example stimulus display where the target is a happy face and distractors are angry faces. Stimuli taken from the NIMH Child Emotional Faces Picture Set [76]

A VST [23] was administered during ET to assess AB for sad and angry faces with a RT- and an ET-based measure within the same task. Each trial started with a drift correction (small white circle in the center of the screen). Upon fixation of the circle, the experimenter initiated the trial. A fixation cross was then presented for 500 ms in the middle of the screen. Subsequently, the stimuli were presented in a 4 × 4 grid containing 15 distractors and one target (Fig. 2). Each stimulus display contained all 16 models randomly allocated to the 16 positions in the grid. The participants’ task was to identify the face showing a certain emotion (i.e., the target) as quickly as possible and click on it with the mouse. Time to identify the target face was not limited. The experiment consisted of four blocks: Two in which happy faces were targets and negative (either sad or angry) faces served as distractors and two in which negative faces (either sad or angry) were targets and happy faces were the distractors. Each block comprised 32 trials with the target appearing twice in each position and being twice each model. The order of trials within the blocks as well as the order of blocks was random. Before each block, participants completed three practice trials to familiarize themselves with the task.

#### Passive-Viewing Task (PVT)

**Fig. 3:**
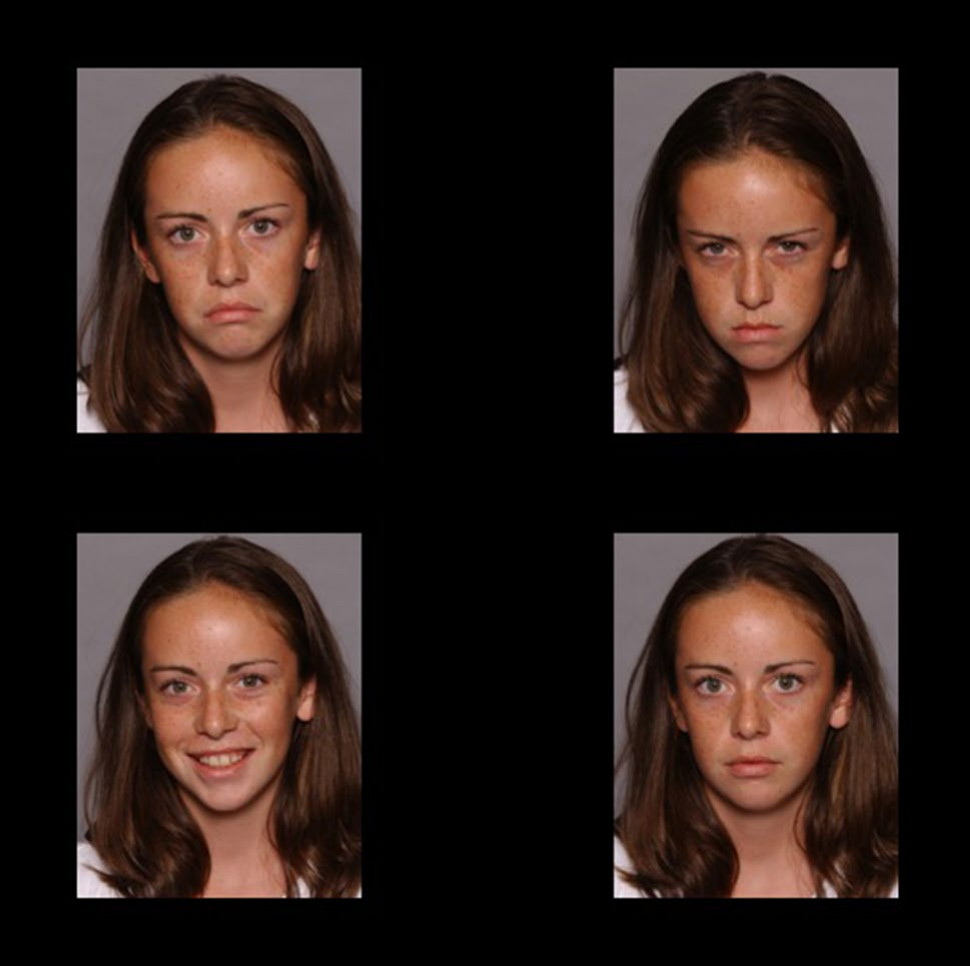
An emotional trial of the children’s version of the Passive Viewing Task (PVT) [50]. Stimuli taken from the NIMH Child Emotional Faces Picture Set [76]

As a purely ET-based measure of AB, a PVT [50] was also administered. Each trial began with a drift correction. A fixation cross followed for 1000 ms. Then the 2 × 2 stimulus array was presented for 15000 ms. The task consisted of 16 emotional trials (corresponding to the minimum trial number suggested for ET research suggested by Orquin and Holmqvist [80]) and eight neutral trials (not analyzed) that were presented in random order. In the emotional trials, the stimulus array comprised four photographs of the same model displaying a sad, an angry, a happy, and a neutral facial expression (see Fig. 3). The position of each emotional facial expression was randomly assigned to one of the quadrants with each emotion being presented in each quadrant exactly four times. The neutral filler trails comprised four photographs of the same person with a neutral facial expression. Stimuli had a size of approximately 9.5 cm × 7.5 cm and were presented with a distance of approximately 6.5 cm horizontally and 1 cm vertically between them. Participants were instructed to first fixate on the white circle and the fixation cross and then freely view the stimuli with the only requirement being that their attention had to remain on the screen.

### Self-Report Measures

#### Symptoms of Depression

The German version of the Children’s Depression Inventory (DIKJ) [81] and the German version of the Beck Depression Inventory-II (BDI-II) [82] were administered to assess depressive symptoms in children and parents. A score for depressive symptoms was available for 79 of the 80 children and 78 of the 80 parents; reliability was good in the current sample (DIKJ: Cronbach’s α = .83; BDI-II: Cronbach’s α = .92).

#### Symptoms of Anxiety

Anxiety was measured by the trait scales of the German version of the State Trait Anxiety Inventory for Children (STAIC) [83] in children and the German version of the State Trait Anxiety Inventory (STAI) [84] in parents. Anxiety scores were available for all children and 78 of the 80 parents and reliability in the current sample was good (STAIC-T: Cronbach’s α = .83; STAT-T: Cronbach’s α = .94).

### Eye-Tracker

During the PVT and VST, eye movements were registered with an EyeLink 1000 Plus Desktop mounted eye-tracker which uses infrared video-based tracking technology (SR research). Participants were seated in front of a 15-inch monitor (1024 × 768 pixel resolution) on which the experiments were presented using Experiment Builder 1.10 (SR Research, 2013). Viewing was binocular while eye movements were registered from the dominant eye with a sampling rate of 1000 Hz. A forehead and chin rest were used to minimize head movements and keep the viewing distance constant at 65 cm. Lighting of the room was kept constant for all participants. Before each task started, a 9-point calibration and validation procedure was conducted and calibration was accepted if the average error was less than 0.5° of visual angle and the maximum error was less than 1° of visual angle.

Eye movement events were detected using a velocity and acceleration based saccade detection method with saccades defined, in line with other studies [e.g., 13, 85], as events with a velocity above the threshold of 30°/s or an acceleration above the threshold of 8000°/s^2^. Gaze positions that were stable within 1° of visual angle for at least 100 ms were defined as fixations (in line with other studies [e.g., 86]).

To ensure adequate data quality, ET data of each participant were visually inspected for systematic calibration errors. Trials in which the total dwell time was less than 75% of the presentation time (due to excessive blinks, missing data, or participants not looking at the screen [85]) were excluded. Subsequently, participants with less than 70% valid trials [86] and participants with systematic calibration errors were excluded from the analysis of the ET data.

### Procedure

The present study was part of a larger project which also included tasks assessing IB [66]. In total, children completed six tasks while parents completed five tasks.^12^ Children and parents were tested simultaneously, with tasks presented in a random order. The course of the experimental session corresponds to that of Sfärlea et al. [67 Supplement 5]. A mood induction procedure was administered twice during the experimental session: participants watched a 2 min scene from the movie *The Lion King* [87] that successfully induced unpleasant mood in adults and children in earlier studies [88, 89] as well as ours: both parents and children reported a significantly worse mood (assessed using the valence dimension of the 9-point Self-Assessment Mannequin scale [90]) after watching the movie scene compared to baseline (*t*s ≥ 7.6; *p*s < .001). Details are presented in Supplement 2.

### Data Analysis

Statistical data analysis was conducted with SPSS 25 [91]. For all analyses, the significance level was set to *p* = .05 (two-tailed) and adjusted according to the Bonferroni procedure when multiple post-hoc comparisons were performed.

#### Data Processing and Outcome Variables

For the DPT and VST, trials with incorrect responses or RTs shorter than 200 ms or longer than 2 SDs above each participant’s mean were excluded, in line with previous studies [e.g., 51]. Then, participants with poor accuracy (a correct and valid trial rate of 2 SDs below the mean rate of children or parents) were identified as outliers in terms of accuracy and excluded from the analysis of that particular task.^13^ In the remaining sample of 75 children and 74 parents for analysis of the DPT data, on average 162.3 correct and valid trials per participant were available for the children (*SD* = 17.9; 81% of 200 trials; not different between groups; *t* < 1) and 177.6 trials for the parents (*SD* = 10.7; 89% of 200 trials).^14^ For the VST, a sample of 77 children (with on average 120.6 correct and valid trials per participant; *SD* = 1.8; 94% of 128 trials) and 77 parents (with on average 121.7 correct and valid trials per participant; *SD* = 1.4; 95% of 128 trials; not different between groups in both children and parent samples, *t*s ≤ 1.1, *p*s > .1) was available for analysis of the behavioral data. For the analysis of the VST ET data we additionally excluded trials with poor ET data quality and participants with insufficient trials available^15^ (see “Eye-Tracker” for criteria) resulting in a sample of 74 children and 77 parents with on average 116.2 trials (*SD* = 5.6; 91% of 128 trials) per participant available for the children and 120.9 trials (*SD* = 1.9; 94% of 128 trials) for the parents (not different between groups, *t*s < 1).

For the DPT, AB scores (AB_DPT_) were calculated by subtracting the mean RT in congruent trials from the mean RT in incongruent trials [51, 92], so that positive values indicate an AB towards negative information while negative values indicate an AB away from negative information. Similarly, for the VST, behavioral AB scores (AB_VST-RT_) were calculated by subtracting the mean RT in blocks with negative targets and positive distractors from the mean RT in blocks with positive targets and negative distractors [23, 92], so that positive values indicate more interference by negative information (i.e., a negative AB) and negative values indicate more interference by positive information (i.e., a positive AB). Analogous scores were calculated for the VST ET data (AB_VST-ET_) by subtracting the mean percentage of dwell time on positive distractors from the mean percentage of dwell time on negative distractors. Separate AB scores were computed for sad and angry faces.

Regarding the PVT, after discarding low data quality trials and excluding participants with an insufficient number of trials available due to data quality,^16^ a sample of 75 children (with on average 15.3 trials available per participant; *SD* = 1.1; 96% of 16 trials) and 74 parents (with on average 15.6 trials per participant; *SD* = 0.9; 98% of 16 trials; not different between groups in both children and parent samples, *t*s ≤ 1.3, *p*s > .1) remained for analysis. Two ET indices were analyzed: percentage of first fixations on each emotion (i.e., initial attention orientation; PVT_ORIENTATION_) and mean percentage of dwell time on each emotion (i.e., maintenance of attention; PVT_MAINTENANCE_). PVT_MAINTENANCE_ was analyzed by splitting the trial into five 3000 ms time intervals, in order to investigate the course in attention deployment over time [50].

#### Hypothesis 1: HR Children Show more Negative AB than LR Children

Groups (HR, LR) were compared on AB_DPT_, AB_VST-RT_ and AB_VST-ET_ using *t*-tests. For PVT_ORIENTATION_ an ANOVA with the within-subjects factor Emotion (4: sad, angry, happy, neutral) and the between-subjects factor Group (2: HR, LR) was conducted. For PVT_MAINTENANCE_, a TimeWindow (5: time window 1–5) × Emotion (4) × Group (2) ANOVA was calculated. Degrees of freedom were adjusted via the Greenhouse–Geisser correction when the assumption of sphericity was violated. As the main focus of the study was to compare HR to LR children, only significant effects involving the factor Group were followed up using post-hoc ANOVAs and *t*-tests.

#### Hypothesis 2: HD Parents Show more Negative AB than ND Parents

The same analysis approach was adopted to compare HD and ND parents on the same outcome variables. In order to rule out the possibility that effects were driven by parents currently experiencing an episode of depression, analyses were repeated excluding the currently depressed parents. As this did not change the pattern of results, results reported are based on the whole parent sample.

#### Hypothesis 3: Parents’ AB will Correlate with that of Their Children

Correlations between child and parent AB scores were computed for all outcome variables. For PVT_ORIENTATION_ and PVT_MAINTENANCE_ separate correlations were conducted for each emotion (sad, angry, happy, neutral).

#### Psychometric Properties of the AB Tasks

Reliability (internal consistency) of the AB_DPT_, AB_VST-RT_ and AB_VST-ET_ scores was assessed by calculating split-half reliabilities (by correlating AB scores based on odd versus even trials [see e.g., 18]). For PVT_ORIENTATION_ and PVT_MAINTENANCE_, split-half reliability was calculated for each emotion (as for this task no AB scores were computed; see [93] for a similar approach). Spearman-Brown-corrected split-half reliability scores are reported to enable comparison with traditional guidelines for interpreting reliability coefficients [94]. To assess convergent validity of the tasks, AB scores from the three tasks (DPT, VST, PVT) were correlated with each other, separately for sad and angry faces. Since this involved 40 correlations in total, a Bonferroni correction was applied, meaning that only correlations exceeding the statistical threshold of *p* ≤ .001 were interpreted. Construct validity of the experimental tasks was assessed by investigating relationships between attention indices and psychopathology, i.e., by computing correlations of AB scores (DPT and VST) and ET indices (PVT) with depression (and anxiety) symptoms. Again, a Bonferroni correction was applied to control for multiple (*N* = 44) tests.

## Results

Bias scores for each group are presented in Table 2 (AB_DPT,_ AB_VST-RT_ and AB_VST-ET_) and Table 3 (PVT_ORIENTATION_ and PVT_MAINTENANCE_).

**Table 2:** AB scores for the DPT and VST

	Children		Parents	
	HR	LR	HD	ND
AB_DPT sad_; *M (SD)*	1.0 ms (32.7)	−1.7 ms (27.1)	−0.7 ms (25.2)	2.9 ms (23.0)
AB_DPT angry_; *M (SD)*	0.7 ms (31.7)	−8.5 ms (20.3)	4.5 ms (24.0)	0.8 ms (14.1)
AB_VST-RT sad_; *M (SD)*	−225.3 ms (1135.7)	−283.8 ms (874.2)	−258.9 ms (654.4)	−404.0 ms (522.3)
AB_VST-RT angry_; *M (SD)*	266.7 ms (1250.2)	159.5 ms (906.5)	−434.8 ms (574.8)	−360.3 ms (364.1)
AB_VST-ET sad_; *M (SD)*	−1.3% (5.8)	−0.7% (4.8)	−2.1% (5.0)	−2.7% (6.1)
AB_VST-ET angry_; *M (SD)*	0.8% (6.9)	0.0% (5.4)	−1.6% (5.0)	−1.0% (3.9)

*HR*  high-risk, *LR* low-risk, *HD* history of depression, *ND* never-depressed, *AB*_DPT_ attention bias score from the Dot-Probe Task, *AB*_VST-RT_ behavioural attention bias score from the Visual-Search Task, *AB*_VST-ET_ eye-tracking attention bias score from the Visual-Search Task

### Hypothesis 1: AB in HR Versus LR Children

*T*-tests revealed no significant differences between HR and LR children: neither on the DPT (AB_DPT_ *t*s ≤ 1.5, *p*s > .1), nor on the VST (AB_VST-RT_ *t*s < 1, AB_VST-ET_: *t*s ≤ 1). For the PVT, the Emotion × Group ANOVA on PVT_ORIENTATION_ yielded no significant effects (*F*s ≤ 1.3, *p*s > .1). The TimeWindow × Emotion × Group ANOVA for PVT_MAINTENANCE_ yielded a significant main effect of TimeWindow (*F*_2.1,151.2_ = 13.6, *p* < .001, *η*_*p*_^*2*^ = .16), a significant main effect of Emotion (*F*_2.0,143.5_ = 7.0, *p* = .001, *η*_*p*_^*2*^ = .09), as well as a significant main effect of Group (*F*_1,73_ = 4.1, *p* = .045, *η*_*p*_^*2*^ = .05), resulting from the HR group dwelling less on faces than the LR group. All interactions were non-significant (*F*s ≤ 1.9, *p*s ≥ .05).

### Hypothesis 2: AB in HD Versus ND Parents

*T*-tests revealed no significant differences between HD and ND parents: neither on the AB_DPT_ (*t*s < 1) nor on the VST (AB_VST-RT_: *t*s ≤ 1.1, *p*s > .1, AB_VST-ET_: *t*s ≤ 1). For the PVT, the Emotion × Group ANOVA on PVT_ORIENTATION_ yielded no significant results (*F*s ≤ 2.4, *p*s ≥ .07). The TimeWindow × Emotion × Group ANOVA for PVT_MAINTENANCE_ yielded a significant main effect of TimeWindow (*F*_1.6,113.7_ = 18.3, *p* < .001, *η*_*p*_^*2*^ = .20), a significant main effect of Emotion (*F*_1.4,102.1_ = 47.2, *p* < .001, *η*_*p*_^*2*^ = .4), and a significant TimeWindow × Emotion interaction (*F*_6.3,453.8_ = 14.2, *p* < .001, *η*_*p*_^*2*^ = .17), while all effects involving Group were non-significant (*F*s ≤ 1.2, *p*s > .1).

**Table 3:** AB scores for the PVT

	Children		Parents	
	HR	LR	HD	ND
PVT_ORIENTATION_;* M (SD)*				
Sad	24.5 (11.2)	24.3 (9.6)	27.4 (13.0)	21.4 (9.7)
Angry	23.6 (11.1)	22.3 (11.3)	26.0 (9.7)	25.0 (9.4)
Happy	25.3 (10.2)	28.0 (10.4)	23.2 (10.6)	27.2 (9.1)
Neutral	26.6 (12.0)	25.4 (12.7)	23.5 (12.5)	26.4 (10.6)
PVT_MAINTENANCE_; *M (SD)*				
Sad	20.8 (4.9)	22.9 (3.7)	18.5 (5.0)	19.7 (5.9)
Angry	21.2 (5.8)	22.3 (3.6)	18.7 (4.9)	18.1 (5.5)
Happy	24.0 (7.1)	26.2 (7.8)	33.0 (12.6)	35.6 (13.1)
Neutral	21.9 (5.4)	21.9 (3.1)	24.2 (7.2)	22.9 (6.4)

*HR* high-risk, *LR* low-risk, *HD* history of depression, *ND* never-depressed, *PVT*_ORIENTATION_ % of first fixations in the Passive-Viewing Task, *PVT*_MAINTENANCE_ Mean % of dwell time (averaged across time windows) in the Passive-Viewing Task

### Hypothesis 3: Transgenerational Association between Parent and Child AB

No significant correlations between child and parent AB were found: neither for the AB_DPT_ nor the AB_VST-RT_ or AB_VST-ET_ and regardless of the emotion of stimuli (sad or angry; all |*r*s|≤ .08). Correlations between children’s and parents’ PVT_ORIENTATION_ and PVT_MAINTENANCE_ scores were larger but still non-significant (|*r*s|≤ .24, *p*s ≥ .050).

### Psychometric Properties of the Experimental Tasks

#### Reliability

Split-half reliabilities of the AB_DPT_, AB_VST-RT_, and AB_VST-ET_ scores are presented in Table 4. Reliability of the AB_DPT_ and the AB_VST-ET_ scores was unacceptable (*REL*< .1) in both children and parents. The reliability of the AB_VST-RT_ was acceptable for sad and angry faces in children. In parents the reliability of the AB_VST-RT_ was questionable for sad faces and unacceptable for angry faces. Reliability of the PVT_ORIENTATION_ was unacceptable (across all emotions in children and parents). while reliability of the PVT_MAINTENANCE_ was good to excellent for parents and questionable to good in children (see Table 5).

**Table 4:** Spearman-Brown-corrected split-half reliability scores for AB scores from the DPT and VST

	Children		Parents	
	Sad	Angry	Sad	Angry
AB_DPT_	.04	−.20	.10	−.22
AB_VST-RT_	.73***	.77***	.68***	.43*
AB_VST-ET_	.36	.46*	.52**	.04

*AB*_DPT_ attention bias score from the Dot-Probe Task, *AB*_VST-RT_ behavioural attention bias score from the Visual-Search Task, *AB*_VST-ET_ eye-tracking attention bias score from the Visual-Search Task, *Correlation significant with *p* < .05, **Correlation significant with *p* < .01, ***Correlation significant with *p* < .001

**Table 5:** Spearman-Brown-corrected split-half reliability scores for the PVT

	Children				Parents			
	Sad	Angry	Happy	Neutral	Sad	Angry	Happy	Neutral
PVT_ORIENTATION_	−.13	−.22	−.35	−.17	.11	−.11	−.38	.31
PVT_MAINTENANCE_	.69***	.71***	.84***	.68***	.88***	.89***	.95***	.86***

*PVT*_ORIENTATION_ % of first fixations in the Passive-Viewing Task, *PVT*_MAINTENANCE_ Mean % of dwell time (averaged across time windows) in the Passive-Viewing Task, ***Correlation significant with *p* < .001

#### Convergent Validity

Correlations between the different measures of AB for children and parents are presented in Tables 6 and 7.

**Table 6:** Correlations between different measures of AB in children

	AB_DPT sad_	AB_VST-RT sad_	AB_VST-ET sad_	PVT_ORIENTATION sad_
AB_VST-RT sad_	.01			
AB_VST-ET sad_	.05	.69***		
PVT_ORIENTATION_ _sad_	.00	.10	.13	
PVT_MAINTENANCE_ _sad_	.02	−.01	−.03	.18

	AB_DPT angry_	AB_VST-RT angry_	AB_VST-ET angry_	PVT_ORIENTATION_ _angry_
AB_VST-RT angry_	−.37**			
AB_VST-ET angry_	−.43***	.68***		
PVT_ORIENTATION_ _angry_	−.18	−.10	−.01	
PVT_MAINTENANCE__angry_	−.19	.25*	.13	.19

*AB*_DPT_ attention bias score from the Dot-Probe Task, *AB*_VST-RT_ behavioural attention bias score from the Visual-Search Task, *AB*_VST-ET_ eye-tracking attention bias score from the Visual-Search Task, *PVT*_ORIENTATION_ % of first fixations in the Passive-Viewing Task, *PVT*_MAINTENANCE_ Mean % of dwell time (averaged across time windows) in the Passive-Viewing Task, * Correlation significant with *p* < .05, **Correlation significant with *p*  < .01, ***Correlation significant with *p* < .001

**Table 7:** Correlations between different measures of AB in parents

	AB_DPT sad_	AB_VST-RT sad_	AB_VST-ET sad_	PVT_ORIENTATION sad_
AB_VST-RT sad_	.02			
AB_VST-ET sad_	−.11	.64***		
PVT_ORIENTATION sad_	−.25*	−.10	.09	
PVT_MAINTENANCE sad_	.21	−.06	.10	−.08

	AB_DPT angry_	AB_VST-RT angry_	AB_VST-ET angry_	PVT_ORIENTATION_ _angry_
AB_VST-RT angry_	−.10			
AB_VST-ET angry_	−.05	.52***		
PVT_ORIENTATION_ _angry_	.09	−.03	−.01	
PVT_MAINTENANCE_ _angry_	−.05	.00	.10	.09

*AB*_DPT_ attention bias score from the Dot-Probe Task, *AB*_VST-RT_ behavioural attention bias score from the Visual-Search Task, *AB*_VST-ET_ eye-tracking attention bias score from the Visual-Search Task, *PVT*_ORIENTATION_ % of first fixations in the Passive-Viewing Task, *PVT*_MAINTENANCE_ Mean % of dwell time (averaged across time windows) in the Passive-Viewing Task, * Correlation significant with *p* < .05, *** Correlation significant with *p* < .001

For children as well as parents, correlations between AB_VST-RT_ and AB_VST-ET_ were found for both sad and angry faces (*r*s ≥ .52; *ps* < .001). The only other significant correlation was between the AB_DPT_ and AB_VST-ET_ for angry faces in children (*r* = −.43; *p* < .001).

#### Construct Validity

A few small correlations between symptoms of depression or anxiety and measures of AB emerged (all other |*r*s|< .21, *p*s > .05). In children, depressive symptoms were positively correlated with PVT_*ORIENTATION*_ to happy faces (*r* = .23, *p* = .046) as well as PVT_*MAINTENANCE*_ (averaged across epochs) to sad faces (*r* = .24, *p* = .040). In parents, trait anxiety positively correlated with AB_DPT_ for angry faces (*r* = .28, *p* = .017) and negatively correlated with AB_VST-RT_ for angry faces (*r* = −.23, *p* = .044). None of these correlations survived once a Bonferroni correction was applied.

## Discussion

### Summary of Findings

Negative AB have been implicated in the etiology of adult [8–10] and youth [40, 41] depression, yet their role as a means by which depression risk is transferred from parent to child is unclear. While previous studies suggest negative AB characterize the children of depressed parents, the direction of these effects is unclear. The aim of the current study was to resolve this issue by assessing AB in children of depressed (HR) versus non-depressed (LR) parents using multiple instruments including novel ET methods associated with superior psychometric properties. We also assessed AB in parents with a history of depression (HD) versus those with no psychiatric history (ND) and tested the hypothesis that parent and child AB would be correlated with one another. However, contrary to our expectations, there was no evidence of group differences in AB; neither between HR and LR children, nor between HD and ND parents. Across the sample there was no evidence that parent AB correlated with child AB. Before considering possible explanations for these findings, it is worth first reflecting on the psychometric properties of the tasks in our sample.

### Is there a Reliable Way of Measuring AB?

The finding that the internal consistency of the DPT was unacceptable in both parents and children is consistent with previous studies of adults [13–21] and children [19, 22]. Van Bockstaele, Notebaert, et al. found the VST to be a more reliable measure of AB in unselected adults [24], whereas findings from child samples suggested poorer reliability [19, 22]. Interestingly, in the current study we found the opposite pattern of findings: reliability of the behavioral index of the VST (AB_VST-RT_) was acceptable in children but questionable to unacceptable in parents. The VST was theorized to have better internal consistency because of the longer exposure times in the task (increasing task: error variance). The finding that the VST had poor reliability in the current sample of adults may be explained by the fact that we included parents with a history of depression whereas the previous study of VST reliability recruited undergraduate students without elevated symptoms of psychopathology. It is important that the reliability of tasks is considered in relation to specific populations since mental disorders such as depression are known to influence reaction times. Similarly, the poorer reliability of the VST in previous studies of children may be explained by the fact these studies included younger children (aged 7–9 years) whereas children in the current study were older (9–14 years). Contrary to expectations that ET measures would be associated with superior reliability, the reliability of the ET index of the VST and the PVT orientation index also showed unacceptable reliability. The PVT maintenance index showed superior reliability: reliability in the child sample was questionable to good, and in the parent sample it was good to excellent. The fact that reliability was higher for the maintenance (versus orientation) index corresponds with other reports suggesting that the ET reliability is higher for indices measured over longer (versus shorter) periods of time [25]. In summary, whilst the DPT showed poor reliability in our study, behavioral indices of the VST task along with the maintenance index of the PVT showed better reliability. Whereas the poor reliability associated with AB tasks may reflect methodological limitations of the tasks themselves, others have argued that AB itself may be an unreliable construct, varying within individuals within short periods of time [95].

### Do Children of Depressed Parents Show a more Negative AB?

In light of the poor reliability and construct validity (no correlations with depressive symptoms in children or parents) of the DPT in the current study, it is perhaps not surprising that the task failed to differentiate between HR and LR children. Nevertheless, our findings contradict those of many other studies using similar methodology and samples [54–60] which is hard to explain. It is theoretically possible that the DPT showed superior reliability in previous studies compared to ours, however the psychometric properties of the task in these studies are not reported and given that numerous studies have reported poor reliability of the task in adult [13–21] and child [19, 22] samples, superior reliability is unlikely. Our null-findings are also unlikely to reflect a lack of power, since our sample size is comparable, if not larger, than most previous studies’. For example, one study that found daughters of depressed mothers to show more negative AB than daughters of never-disordered mothers included just 20 HR children [54]. Other studies that had sample sizes comparable to ours (36 and 38 HR children [55, 56]) found negative AB only in HR children who were female [55] or had a parent with AB away from positive information [56]. Two studies that investigated larger samples (241 and 244 HR children respectively [57, 58]) did also not find significant main effects of group but more negative AB only in sub-groups of HR children that showed elevated cortisol reactivity to stress [58] or were female [57]. Unfortunately, our sample was too small to explore whether gender or direction of parental AB might explain our null-effects. Of course one possible explanation is that a publication bias exists in the field of AB in youth depression. Whilst it is common knowledge that small sample sizes reduce the chances of finding a true effect (Type II error), it is less well known that they also result in over-inflated effect sizes due to only large effects passing statistical thresholds [96]. To this extent, it is possible that other studies in children of depressed parents with larger sample sizes have been conducted but failed to be published due to their lack of significant effects. A meta-analysis of AB in at-risk and depressed youth which estimates the likelihood of a publication bias in the field could inform this hypothesis and provide a valuable contribution to future research. In summary, based on the poor reliability of the DPT in our sample and the modest sample sizes of previous studies, we urge extreme caution in the interpretation of their findings.

The null-findings between HR and LR children from the AB_VST-ET_ index and the PVT orientation index can also plausibly be attributed to the poor psychometrics of the tasks in the current sample. However, the null-effects in relation to the AB_VST-RT_ index in HR versus LR children cannot since reliability of this index was acceptable. For the PVT maintenance index, reliability was questionable to good, yet there was no evidence of AB in HR versus LR children. It is worth mentioning that a main effect of group was observed for the maintenance index: HR children spent less time looking at the faces (regardless of emotion) than LR children. This may reflect an avoidance-based emotion regulation strategy which HR children have developed as a result of potentially inconsistent emotions expressed by their parent. Nevertheless, this finding was not predicted and its interpretation remains speculative. It is worth considering whether other methodological factors could explain the null-findings in HR and LR children in the AB_VST-RT_ and PVT maintenance indices. Firstly, the findings cannot be attributed to difficulties children had in completing the tasks since the accuracy rate for the VST was 94% and the PVT did not involve any active response. A second possibility is that children were not in enough of a negative mood state for AB to be observed (cognitive models propose that cognitive biases only emerge under conditions of stress or negative mood [61, 62]). However, the current data suggest that the mood induction was successful in inducing a negative mood. Could the null findings in the HR versus LR children relate to sample characteristics? Since some of the HR children were recruited through a preventive intervention trial, it is theoretically possible that the intervention positively influenced AB in the HR group. However, given that just 10 of the children had participated in the intervention by the time they took part in the present study, this also seems an unlikely explanation for our findings. More plausible is perhaps that children whose parents had the motivation to sign up to an extensive intervention (see footnote 6) despite having a history of depression are less vulnerable to depression in the first place than children of depressed parents who do not sign up for such an intervention. Nevertheless, the fact that group differences (HR versus LR) have been observed for an implicit measure of IB in the same sample [67], the current null findings are unlikely to be due to sample characteristics.

One additional explanation for the lack of differences in AB between HR and LR children might be that AB are rather correlates of depressive symptomatology that arise as a consequence of the disorder rather than antecedents that act as cognitive vulnerabilities or risk factors contributing to the development of the disorder. This might explain why our results are not in line with studies that also included youth with elevated levels of depression in their HR group [52, 59, 60]. Once established, AB may exacerbate depressive symptoms and are likely to contribute to the maintenance of the disorder [97]. To date, only few studies have investigated to what extent cognitive biases are risk factors for depression vs. consequences of depression. Studies on memory biases [98] and IB [99] have found negative biases to be present in both depressed as well as at-risk youth compared to low-risk youth, but to be more pronounced in depressed compared to at-risk youth. However, regarding AB we found in a subsequent study [100] evidence for currently depressed youth to dwell longer on disorder-specific emotional information (i.e., sad faces) than healthy youth, particularly healthy youth at high risk for depression, suggesting that HR youth might even show AB in opposite directions to depressed youth.

### Do Parents with a History of Depression Show a Negative AB?

Although one previous study failed to find evidence of increased attention to negative stimuli in remitted depressed adults [101], the current findings contrast with the majority of previous studies which have found negative AB in adults with a current episode [30] or past history [102–105] of depression. This is perhaps unsurprising given that the tasks generally showed poor reliability in parents. However, the lack of effects in relation to the maintenance index of the PVT was unexpected since reliability of this task was good to excellent. The null-results might be explained by the fact that almost all parents in the HD group had previously received psychotherapy, mostly CBT, which is known to modify cognitive biases [106] and might therefore have reduced their AB. This may be particularly true in situations where conscious processing and intrinsic guidance of attention is possible, as in the PVT. Another possibility might be that some parents were taking psychotropic medication, which is known to influence AB [107]. We exploratorily compared AB scores of HD parents who were taking psychotropic medication with those of HD parents who were not. Since no differences emerged (all *p*s > .1) this is unlikely to account for the null effects. It is unlikely that participation in the preventive intervention through which some participants were recruited accounted for the null-effects, since the intervention targeted parenting strategies but was not designed to modify parents’ symptoms of depression per se. Finally, the severity of parents’ depression (or anxiety) symptoms is unlikely to account for the null-effects, since there was no evidence that any of the AB indices correlated with BDI (depression) or STAI (anxiety) scores. Unfortunately the study was not sufficiently powered to investigate whether single-episode (*n* = 8) versus recurrent (*n* = 34) depression could explain the null effects. Nor whether comorbid disorders (*n* = 12) versus no comorbidity (*n* = 32) could explain the effects. Since the diagnostic status of the second parent was not systematically assessed in the study it was also not possible to explore whether the null-effects were due to having one versus two parents affected by depression.

### Are Child and Parent AB Correlated?

A key hypothesis was that child and parent AB would be associated with another. However, there was no evidence of this for any of the measures. This finding was unexpected since a previous DPT study found AB in parents with a lifetime emotional disorder and their children to be related [56]. However, there are numerous methodological differences between the latter study and our study which make comparisons difficult. For example, in the latter study the majority of parents had an anxiety disorder and only 14 had depression, thus it is possible the positive child-parent association was driven by parents with an anxiety disorder. Secondly, in the latter study both children and parents viewed adult facial stimuli whereas in the current study facial stimuli were matched for age (i.e., children viewed child stimuli). Thus it is possible that our null effects are due to AB being specific to the age of the models. Finally, the robustness of the findings from the study of children of parents with lifetime emotional disorder is questionable: a parent–child AB correlation was only found in the 38 HR children (but not the 29 LR children), and only between child negative AB and parent positive AB (negative correlation) but not parent negative AB. Given the relatively small number of HR children and the specificity of the findings to one form of parent AB, we urge caution in the interpretation of these effects. In fact, our null-effects are also in line with a study of AB in anxious parents and their offspring: There was no evidence for a correlation between parent AB and child AB [19]. Finally, the current findings are also somewhat consistent with additional findings from the same sample in relation to measures of IB [67]: Although HR children and their HD parents showed an implicit negative IB, there was no evidence of transgenerational correlation in this IB. Nevertheless, the fact that we found no evidence of AB in HD parents limits the conclusions that can be drawn from the current study about the transgenerational role of AB.

### Strengths and Limitations of Study

A major strength of the current study is the inclusion of multiple measures of AB; both behavioral (DPT, VST) and ET-based (VST, PVT). Findings from previous studies of AB in children of depressed parents have been difficult to compare due to heterogeneity in their methodology. In this study, consistent null-results across three different tasks enable us to draw conclusions with more certainty and to directly compare the psychometric properties of the various measures within a single sample. A second strength is the use of standardized diagnostic instruments to categorize parental and child psychopathology. Evaluating parents’ psychiatric status via standardized clinical interviews is a more valid means of assessment than self-report [108]. Standardized clinical interviews were administered not only to all participating children and parents but also to the second parent in the ND/LR families to ensure that neither of the child’s parents had a history of depression or any other psychiatric disorder. Finally, the study makes important contributions towards the open science movement (https://www.cos.io/). Recommendations for addressing the replication crisis in psychology include pre-registration, full-disclosure of analysis methods and publication of non-significant findings [109, 110]. This study was registered on the OSF prior to data collection. It is the first study of AB in children of depressed parents, and one of very few of youth in general [19, 22], to report the psychometric properties of the experimental tasks employed. In contrast with most ET studies, we report data pre-processing steps and the data analysis strategy in great detail. Finally, in light of the “filedrawer” problem, and publication bias the publication of non-significant findings makes a valuable contribution to the existing literature.

Some limitations of the study are also important to mention. The relatively modest sample size means that we cannot exclude the possibility that our null-effects are due to a lack of statistical power. Although we based our sample size calculation on previous studies using similar methodology in the same population [54, 55], in hindsight these effects are likely to have been over-estimates of the true effect. Unfortunately, we had no meta-analysis of AB effect sizes in youth depression to guide our sample size calculation. A second limitation of the sample is the fact that some parents in the HD group and their HR children were recruited via an ongoing preventive intervention for families affected by depression and may therefore be unrepresentative of families with a depressed parent in general. However, as mentioned above, this is unlikely to be enough of an explanation for our null-results. Finally, whilst ET methodology has many advantages over behavioral measures of AB, it does also carry some limitations [25]. Firstly, the psychometric properties of ET measures are not always superior to RT measures and are under-investigated in many populations (e.g., in children and adolescents). Secondly, it must be acknowledged that it cannot detect changes in covert attention since covert attention can occur without eye movements. Nevertheless, covert attentional processes are largely thought to mediate overt attentional processes.

### Clinical Implications

Given the lack of an association between parent and child AB, it is unlikely that AB plays a role in the transmission of depression risk from parent to child. In line with findings on the association between parent and child IB in the same sample [67], it is possible that whilst reflective cognitive factors are passed on from parent to child, implicit processes are not. Our findings provide further evidence that the validity of apparently positive findings from previous DPT studies of AB in HR children and their parents be called into question [54–60]. Since just one study has shown evidence of cross-sectional parent–child associations of AB and no study has yet examined the role of AB in the onset of depression prospectively, we are far from implicating AB in the transgenerational transmission of depression risk. A recent meta-analysis demonstrates a clear need for improved preventive interventions for the children of depressed parents [111]. In the increasing popularity of cognitive bias modification of attention (CBM-A) paradigms, the current study suggests the field is not ready for CBM-A interventions for the children of depressed parents. Similarly, although the DPT has been used to assess changes in AB following therapeutic interventions, the poor psychometric properties generated in this study suggest this is an inappropriate task for such purposes.

### Future Research

The focus of AB research in the field of depression has largely been on biases towards *negative* information. However, recent meta-analyses demonstrate that depression is also characterized by an avoidance of *positive* stimuli [112]. It seems plausible that avoidance of positive stimuli may be involved in the transgenerational transmission of depression, and this may be a valuable area of future research. Based on the findings of the current study, other researchers are urged to evaluate and report the psychometric properties of the tasks they use to investigate AB. The poor psychometric properties of the DPT observed in this study combined with the lack of reporting on psychometric properties in previous studies of children of depressed parents suggests researchers should be cautious about using this paradigm in this sample. Note that one study found that the poor reliability of the DPT is in part influenced by issues to do with data preparation and analysis, which can be optimized to achieve more moderate reliability [113]. However, as others note [65], even when these recommendations are followed, reliability does not necessarily improve [114]. As such, there is also a need for alternative measures of AB to be developed with improved psychometric properties that also adequately address the natural variability of the construct AB itself [95]. Although ET measures were expected to show superior reliability to the DPT, this was not entirely the case, suggesting that one should not assume ET indices to be necessarily superior. A valuable line of future research would also be to develop more reliable ET measures of AB.

Of note, we observed some developmental differences in task reliability: the maintenance index of the PVT showed better reliability in parents than children whereas the behavioral VST index showed better reliability in children than parents. Future studies might involve adapting existing tasks to improve reliability within age groups. A related avenue of future research is to develop a clearer criteria as to what constitutes acceptable reliability for experimental tasks [13]. Once AB can be reliably measured and observed in the children of depressed parents, an important next step would be not only to assess correlations between parents and children cross-sectionally [56] but also longitudinally [115]. Such studies are in a better position to determine the extent to which AB observed in HR children are responsible for the increased onset of depression.

## Summary

The current study sought to investigate whether negative AB may be a possible candidate for the transfer of depression risk from parent to child. Contrary to expectations, there was no evidence of AB in HR versus LR children and no evidence of a correlation between parent and child AB. This may be in part due to the psychometric properties of the measures employed but cannot alone explain these findings. Caution is therefore urged in the inclusion of AB in models of the transgenerational transmission of depression risk. Whereas the DPT showed very poor reliability, ET indices of AB that were measured over a longer period of time in the PVT showed more favorable psychometric properties. It is recommended that future studies thoroughly evaluate and disseminate the psychometric properties of their tasks.

## Supplementary Information

Below is the link to the electronic supplementary material.

Supplementary Information 1 (DOCX 30 kb)

## Footnotes

1 Other behavioral measures of AB are the Emotional Stroop Task (EST) [48], the Go/No-Go Task [116] and Posner’s Spatial Cueing Paradigm [117]. Whilst the EST was used in early studies, it might tap attention suppression rather than orientation, as suggested by the poor correlation between EST and other AB tasks [118].

2 Traditional guidelines for interpreting reliability estimates (*REL*) as are follows: *REL* < .50 “unacceptable”; *REL* = .5-–6 “poor”; *REL* = .6-–7 “questionable”; *REL* = .7-–8 “acceptable”; *REL* = .8-– “good”; *REL* > .9 “excellent” [94].

3 Throughout the manuscript Sawilowsky ‘s recommendation for the interpretation of effect sizes (ES) are used: 0.01 (very small), 0.20 (small), 0.50 (medium), 0.80 (large), 1.20 (very large), 2.0 (huge) [119].

4 Participants also completed the Scrambled Sentences Task (SST) [120] during ET (as adapted by [121]) in order to assess IB (behavioural results) and AB (through ET) simultaneously. Due to technical difficulties parent ET data could not be analysed therefore child AB results are presented in Supplement 1.

5 81 dyads were tested but one family was excluded due to bad compliance. One HR child was accompanied by the non-depressed parent so data from the experimental tasks are not available for this parent.

6 Families were approached either (i) immediately after baseline assessment for the prevention trial, (ii) at a post-intervention assessment, or (iii) somewhere in between. They were asked if they were interested in participating in the current study and given information material. Of the 28 HD/HR-families recruited through the prevention trial, 10 had already participated in the intervention by the time they took part in the present study while the others took part before receiving the intervention. The family- and group-based cognitive behavioural intervention for children of parents with a history of depression consisted of 8 weekly sessions and 4 monthly booster sessions. The intervention targeted children’s coping with stress (e.g., positive thinking and distraction) and parenting skills in parents, as well as family interaction.

7 Families that had participated in previous studies received letters inviting them to participate in the present study, including information material on the project. Similar letters were sent to families with children in the corresponding age range whose addresses were provided by the local registry office. Those were randomly chosen from all families in Munich with children in the corresponding age range.

8 Analyses excluding these two families revealed the same pattern of results.

9 via the DIPS (available from 75% of families) and BDI-II [82] (available from 81% of families; *M* = 1.3, *SD* = 3.1 in the second parent).

10 One girl in the HR group met criteria for enuresis in the past but was included nonetheless.

11 Of the HD parents, 30 reported no symptoms of depression currently, six reported mild depression symptoms, six reported moderate depression symptoms, and two reported severe depression symptoms (according to BDI-II clinical cut-offs: 0–12 no depression; 13–19 mild depression, 20–28 moderate depression, 29–63 severe depression [82]). Of the ND group, 32 reported no symptoms of depression and one reported mild depression symptoms.

12 Children completed the 3 tasks assessing AB presented here (DPT, VST, PVT), 2 tasks assessing IB presented elsewhere [67] and the SST assessing IB and AB simultaneously (see [67], footnote 4, and Supplement 1). Parents completed the 3 tasks assessing AB presented here, 1 task assessing IB presented elsewhere [67] and the SST assessing IB and AB simultaneously. However, due to technical difficulties parent ET data from the SST could not be analysed; see footnote 4 and [67].

13 DPT: *n* = 4 HR children, *n* = 1 LR child, *n* = 3 HD parents, *n* = 2 ND parents; VST: *n* = 2 HR children, *n* = 1 LR child, *n* = 2 HD parents.

14 Significantly less trials were available for the HD group (*M* = 174.7, *SD* = 11.1) than for the ND group (*M* = 181.1, *SD* = 9.2), *t*_72_ = 2.7, *p* = .009.

15 *n* = 1 HR child, *n* = 2 LR children.

16 Children: *n* = 3 HR, *n* = 1 LR; parents: *n* = 2 HD, *n* = 1 ND; data of 1 h child and 1 HD parent was not available due to technical difficulties.
