# Supplementary material for: An Eye-Tracking Study of Attention Biases in Children at High Familial Risk for Depression and Their Parents with Depression
Source: Child Psychiatry Hum Dev. 2021 Jan 4;53(1):89–108. doi: 10.1007/s10578-020-01105-2 (PMC8813682; doi:10.1007/s10578-020-01105-2)
Supplement: Supplementary file 1 — Supplementary Information 1 (DOCX 30 kb) [file 10578_2020_1105_MOESM1_ESM.docx]

**Supplement 1: Scrambled Sentences Task**

**Method**

A computerized version of the Scrambled Sentences Task (SST; Wenzlaff & Bates, 1998; adapted by Everaert, Duyck, & Koster, 2014) was used to assess the tendency to form negative or positive statements out of ambiguous verbal information. We administered the task during eye-tracking in order to simultaneously assess attention and interpretation biases (Everaert et al., 2014), but data on interpretation biases are reported elsewhere (Sfärlea et al., 2019). The task was administered to both children and parents but due to technical difficulties we could not analyse the parent attention bias data. Therefore, only results of the attention bias in children are presented here.

**Stimuli.** For children, the stimuli consisted of 50 scrambled sentences, of which 30 were emotional sentences (e.g., “total I winner a loser am”) and 20 were neutral sentences (e.g., “like watching funny I exciting movies”). The emotional sentences were based on the original stimulus set developed by Wenzlaff and Bates (1998) which was translated into German (Rohrbacher, 2016), adapted, and extended (an English translation of all sentences is provided in Sfärlea et al., 2019, Supplement 4). Sentences that were easily understandable and relevant to children were chosen. All sentences contained six words and had two possible solutions. In emotional trials, one solution was positive (e.g., “I am a total winner”) whereas the other was negative (e.g., “I am a total loser”). In neutral trials both solutions were emotionally neutral. Across the stimulus set, target words (the words in each sentence that accounted for the positive or negative solution) were matched for length and frequency in the German language.^[[1]](#footnote-1)^ In line with Everaert et al. (2014), word position within each sentence was randomised, with target words not allowed next to each other or in the first or last position and counterbalanced whether the positive or negative target word was presented first.

**Task Procedure.** The trial procedure in depicted in Sfärlea et al. (2019; Figure 2). The experiment was presented using SR Research Experiment Builder 1.10 (SR Research Ltd., 2013). Each trial started with a fixation cross that was presented for 500 ms on the left side of the screen (to elicit left to right reading). This was followed by a stimulus display consisting of six words in a scrambled order presented at the centre of the screen on a single line. Participants were instructed to read the words and mentally form a grammatically correct five-word sentence as quickly as possible and to click on the mouse button as soon as they did so to continue to the response part of the trial. The scrambled sentence was presented for a maximum of 8000 ms, if no mouse click occurred during that time, the response part was omitted and the next trial began. In the response part of the trial five boxes appeared below the scrambled sentence and participants were required to build the sentence they had mentally formed by ordering the words into the five boxes provided. The first mouse click allocated a word to the first box, the second click to the second box and so on. Time to allocate the words to boxes was not limited.

Trials were randomly divided into five blocks of ten (each containing six emotional and four neutral trials presented in random order). Before the first block participants completed five practice trials (five randomly selected neutral sentences) to familiarize themselves with the task.

Similar to earlier studies (e.g., Everaert et al., 2014; Rude, Valdez, Odom, & Ebrahimi, 2003), a cognitive load procedure was added to prevent deliberate report strategies. Before each block, a 4-digit number was presented to the children for 5000 ms. Participants were instructed to memorize the number in order to recall it at the end of the block.

**Data Processing and outcome variables.** Only the emotional trials were analysed. Participants’ responses were rated as correct or incorrect. Trials in which no grammatically correct sentence was built (time-out or incorrect sentence) as well as trials with poor data quality (total dwell time of less than 75% of the presentation time due to excessive blinks, missing data, or participants not looking at the screen; Skinner et al., 2018) were excluded. Two participants with severe reading difficulties were excluded, as they were hardly able to perform the task. In addition, participants of whom less than 70% of correct and valid trials remained available after trail exclusion (e.g., [Duque & Vázquez, 2015](#_ENREF_4)) and participants with systematic calibration errors identified by visual inspection were excluded from the analysis (altogether *n* = 15 HR, *n* = 15 LR; not different between groups, χ² < 1), resulting in a sample of 47 children for analysis of the SST AB data. In that remaining sample, on average 24.8 trials (*SD* = 2.4; 83% of 30 trials) per participant were available (not different between groups; *t* < 1).

Similar to Everaert et al. (2014), we calculated an AB_SST_ score by dividing the percentage of dwell time on negative target words by the sum of percentage of dwell times on negative and positive target words, so that a higher value indicated a more negative AB.

**Data analysis.** A *t*-test was calculated to compare the AB_SST_ between HR and LR groups. Split-half reliability of the SST was calculated by correlating bias scores based on odd versus even trials (see e.g., Van Bockstaele, Salemink, Bögels, & Wiers, 2017). Construct validity was assessed by investigating correlations between AB_SST_ score and psychopathology, i.e., depression and anxiety scores. Convergent validity was determined by correlating the AB_SST_ score with AB scores (DPT and VST) and ET indices (PVT) from the other tasks.

**Results**

**Group comparisons.** The *t*-test revealed that HR (*M* = 49.0%, *SD* = 4.6) and the LR (*M* = 51.6%, *SD* = 7.2) groups did not differ significantly in the AB_SST_ score (*t*_45_ = 1.5, *p* > .1).

**Psychometric properties**. Split-half reliability of the AB_SST_ score was very poor (*r* = -.36; *p* = .012). Regarding construct validity, no correlations between AB_SST_ scores and depression or anxiety symptoms emerged (|*r*s| ≤ .28, *p*s > .05). With respect to convergent validity, all correlations between AB_SST_ and AB from the other tasks were non-significant (*|r*s| ≤ .25, *p*s > .1).

**Discussion**

We found no evidence of an AB in the HR compared to the LR sample. However, interpretation of this result is compromised as several problems with the AB measure derived from the SST have to be noted. First, we had to exclude almost 40% of the participants due to poor ET data quality (i.e., either less than 70% of correct and valid trials available or systematic calibration errors) resulting in a sample of only *n* = 27 HR and *n* = 20 LR participants. Second, psychometric properties of the measure were very poor: the split-half correlation coefficient was even negative.

This contrasts with our results on the interpretation bias measure from this task for which we found fair reliability as well as validity and which differentiated between HR and LR children as well as HD and ND parents (see Sfärlea et al., 2019).

Previous studies using a computerized version of the SST in order to simultaneously assess attention and interpretation biases (e.g., Everaert et al., 2014) have not reported on the psychometric properties of the measures. We therefore recommend future studies using this task to carefully monitor data quality and to asses and report the psychometric properties of the measures, especially the AB measure. The SST might need further improvement in order for the AB measure to be as reliably and valid as the interpretation bias measure.

**References**

Duque, A., & Vázquez, C. (2015). Double attention bias for positive and negative emotional faces in clinical depression: Evidence from an eye-tracking study*. Journal of Behavior Therapy and Experimental Psychiatry*, *46*, 107-114.

Everaert, J., Duyck, W., & Koster, E. H. W. (2014). Attention, interpretation, and memory biases in subclinical depression: A proof-of-principle test of the combined cognitive biases hypothesis. *Emotion, 14,* 331-340.

Rohrbacher, H. (2016). Interpretation bias in the context of depressed mood: Assessment strategies and the role of self-generation in cognitive bias modification (Doctoral Dissertation, TU Chemnitz). Retrieved from <http://nbn-resolving.de/urn:nbn:de:bsz:ch1-qucosa-207298>.

Rude, S. S., Valdez, C. R., Odom, S., & Ebrahimi, A. (2003). Negative cognitive biases predict subsequent depression. *Cognitive Therapy and Research*, *27*, 415–429.

Sfärlea, A., Löchner, J., Neumüller, J., Asperud Thomsen, L., Starman, K., Salemink, E., Schulte-Körne, G., & Platt, B. (2019). Passing on the half-empty glass: A transgenerational study of interpretation biases in children at risk for depression and their parents with depression. *Journal of Abnormal Psychology*, *128*, 151-161.

Skinner, I.W., Hübscher, M., Moseley, G.L., Lee, H., Wand, B.M., Traeger, A.C., Gustin, S.M., & McAuley, J.H. (2018). The reliability of eyetracking to assess attentional bias to threatening words in healthy individuals. *Behavior Research Methods*, *50*(5), 1778-1792.

SR Research Ltd. (2013). SR Research Experiment Builder 1.10. [Computer software]. Mississauga, Canada: Author.

Van Bockstaele, B., Salemink, E., Bögels, S. M., & Wiers, R. W. (2017). Limited generalisation of changes in attentional bias following attentional bias modification with the visual probe task. *Cognition and Emotion*, *31*, 369–376.

Wenzlaff, R. M., & Bates, D. E. (1998). Unmasking a cognitive vulnerability to depression: How lapses in mental control reveal depressive thinking. *Journal of Personality and Social Psychology*, *75*, 1559–1571.

**Supplement 2: Results of the mood induction**

Mood was assessed five times during the experimental session (see Sfärlea et al., 2019, Supplement 5) using the valence dimension of the 9-point Self-Assessment Mannequin scale (Lang, 1980) and analysed for children and parents separately with Time (5) × Group (2) ANOVAs and subsequent *t*-tests.

For the children, the ANOVA yielded a significant main effect of Time (*F*_1.9,146.6_ = 65.1; *p* < .001) but no main effect or interaction of Group (*F*s ≤ 1.2; p > .1). Post-hoc *t*-tests comparing the baseline mood (mood before first mood induction: Time 1) with the other mood assessments revealed that participants rated their mood worse after watching the sad movie scene (Time 2 and Time 3; *t*s ≥ 7.6; *p*s < .001) and better after watching the pleasant movie scene (Time 5; *t*_79_ = 3.4; *p* = .001) compared to baseline (see Supplementary Figure 1).

**Supplementary Figure 1:** Mean mood ratings of the children in each of the five mood assessments. Error bars represent standard deviations.

For the parents, the ANOVA yielded a significant main effect of Group (*F*_1,77_ = 4.5; *p* = .037) indicating that the HD group rated its mood worse than the ND group. In addition, a main effect of Time (*F*_2.0,154.7_ = 61.4; *p* < .001) emerged and was followed up by post-hoc *t*-tests comparing the baseline mood (mood before first mood induction: Time 1) with the other mood assessments: participants rated their mood worse after watching the sad movie scene (Time 2 and Time 3; *t*s ≥ 7.9; *p*s < .001) and better after watching the pleasant movie scene (Time 5; *t*_78_ = 3.7; *p* < .001) compared to baseline (see Supplementary Figure 2).

**Supplementary Figure 2:** Mean mood ratings of the parents in each of the five mood assessments. Error bars represent standard deviations.

**References (Supplement 1 and 2):**

Hahn, D. (Producer), Allers, R., & Minkoff, R. (Directors). (1994). *The Lion King* [Motion Picture]. USA: Walt Disney.

Lang, P. J. (1980). Behavioral treatment and bio-behavioral assessment: Computer applications. In J. B. Sidowski, J. H. Johnson & T. A. Williams (Eds.), *Technology in Mental Health Care Delivery Systems* (pp. 119-137). Norwood: Ablex.

Sfärlea, A., Löchner, J., Neumüller, J., Asperud Thomsen, L., Starman, K., Salemink, E., Schulte-Körne, G., & Platt, B. (2019). Passing on the half-empty glass: A transgenerational study of interpretation biases in children at risk for depression and their parents with depression. *Journal of Abnormal Psychology*, *128*, 151-161.

1. Positive target words: Word length *M* = 7.3 (*SD* = 2.6) characters, word frequency (category according to http://wortschatz.uni-leipzig.de/de) *M* = 10.3 (*SD* = 2.9); Negative target words: word length *M* = 7.4 (*SD* = 2.6) characters, word frequency *M* = 10.3 (*SD* = 4.0); *t*s < 1 in paired *t*-tests. [↑](#footnote-ref-1)
